# *Channa argus* BMH from Baima Hu Lake: sequencing and phylogenetic analysis of the mitochondrial genome

**DOI:** 10.1080/23802359.2020.1775144

**Published:** 2020-06-11

**Authors:** Minyan Liu, Junxia Yin, Jiwei Han, Jiangfeng Ren, Shoubao Yang

**Affiliations:** a College of Life Sciences, Shaoxing University, Shaoxing, P.R. China; b Chunhui Senior High School, Shangyu, P.R. China

**Keywords:** Mitogenome, *Channa argus*, sequencing, phylogenetic analysis

## Abstract

Northern snakehead, *Channa argus*, is a commercially important food fish species in China. In the present study, the complete mitochondrial genome of *C.argus* from the Baima Hu Lake was characterized. It is 16,558 bp in length, consist of 22 tRNA genes, 13 PCD genes, 2 rRNA genes, and 1 D-loop region. The overall base composition of the *C. argus* mitogenome is 27.26% A, 24.21% T, 31.58% C and 16.95% G, exhibits a similar AT bias (51.47%) feature to other vertebrate mitogenomes. The phylogenetic analysis showed that *C. argus* clustered in genus *Channa*. The present resultes provide useful information to population genetics and conservation biology studies of *Channa* fishes.

Northern snakehead *Channa argus*, a benthic carnivorous fish (Yue et al. 1996; Ermolenko and Besprozvannykh 2008; Dong et al. 2014a; Duan et al. 2018), which is distributed widely in various water systems of China, North Korea, Japan, Southeast Asia, India and Russian (Odenkirk and Owens 2005; Hossain et al. 2008; Nguyen et al. 2012; Dong et al. 2014b; Densmore et al. 2016). It is a commercially important fish species in China known for its fast growth, high meat content with few bone spurs, tolerance to water pollution and diseases (Ishimatsu and Itazawa 1983; Sagada et al. 2017; Zhou et al. 2018; Chen et al. 2019; Li et al. 2019; Fang et al. 2019).

The Baima Hu Lake is a small lake in Shangyu district, Shaoxing city, East China. Although *C.margus* is artificially cultured for a long time in China (Wang et al. 2012, 2019), little is known about it in the Baima Hu Lake.

The mitogenome could provide useful data for population genetics and conservation biology studies of vertebrate fish, due to rich signals from its sequence and conserved gene arrangement (Liu and Cui 2009; He et al. 2014; Li et al. 2016; Zhu et al. 2019).

Herein, the complete mitogenome of *C. argus* (GenBank accession no. MN781664) was characterized. *Channa margus* individual was sampled from the Baima Hu Lake, Zhejiang Province of China (33°13′47.7″N 119°08′49.4″E), and was kept in 99% ethanol in the Aquatic Service Platform of Shaoxing (accession no. SXAF20200508). The PCR fragments were amplified and sequcenced.

The complete mitochondrial genome of *C. argus* is 16,558 bp in length, consists of 22 tRNA genes, 13 protein-coding genes (PCDs), 2 rRNA genes, and 2 non-coding regions. The total length of the protein-coding gene sequences is 11,900 bp. Except for the ND6 being encoded on the L-strand, all the other PCD genes (ND1-5, ND4L, COXI-III, ATP6, ATP8, and CytB) are encoded on the H-strand. The total length of all tRNA genes is 1540 bp, varying from 65 bp (tRNA^Cys^) to 75 bp (tRNA^Lys^). The 12S rRNA gene (946 bp) and 16S rRNA (1687 bp) gene are located between two tRNA genes (tRNA^Phe^ and tRNA^Leu(UUR^), and are separated by tRNA^Val^ gene. The length of D-loop region is 992 bp. The gene structure and arrangement of *C. argus* are very similar to other vertebrate species (Chen et al. 2013; Yang et al. 2015).

The overall base composition of the *C. argus* mitogenome is 27.26% A, 24.21% T, 31.58% C and 16.95% G, respectively, which exhibits a similar AT bias (51.47%) feature to other vertebrate mitogenomes (Yang et al. 2015; Cui et al. 2019).

The phylogenetic analysis showed that *C. argus* is clustered with other *C. argus* reported (Wang and Yang 2011), and clustered together with other *Channa* fishes, including *Channa asiatica*, *Channa diplogramme*, *Channa gachua*, *Channa lucius*, *Channa maculata*, *Channa marulius*, *Channa striata*, and *Channa punctata* (Figure 1). But, it showed distant kinship with other Cyprinidae fishes. This study provides useful data to population genetics and conservation biology studies of Channa fishes.

**Figure 1.**
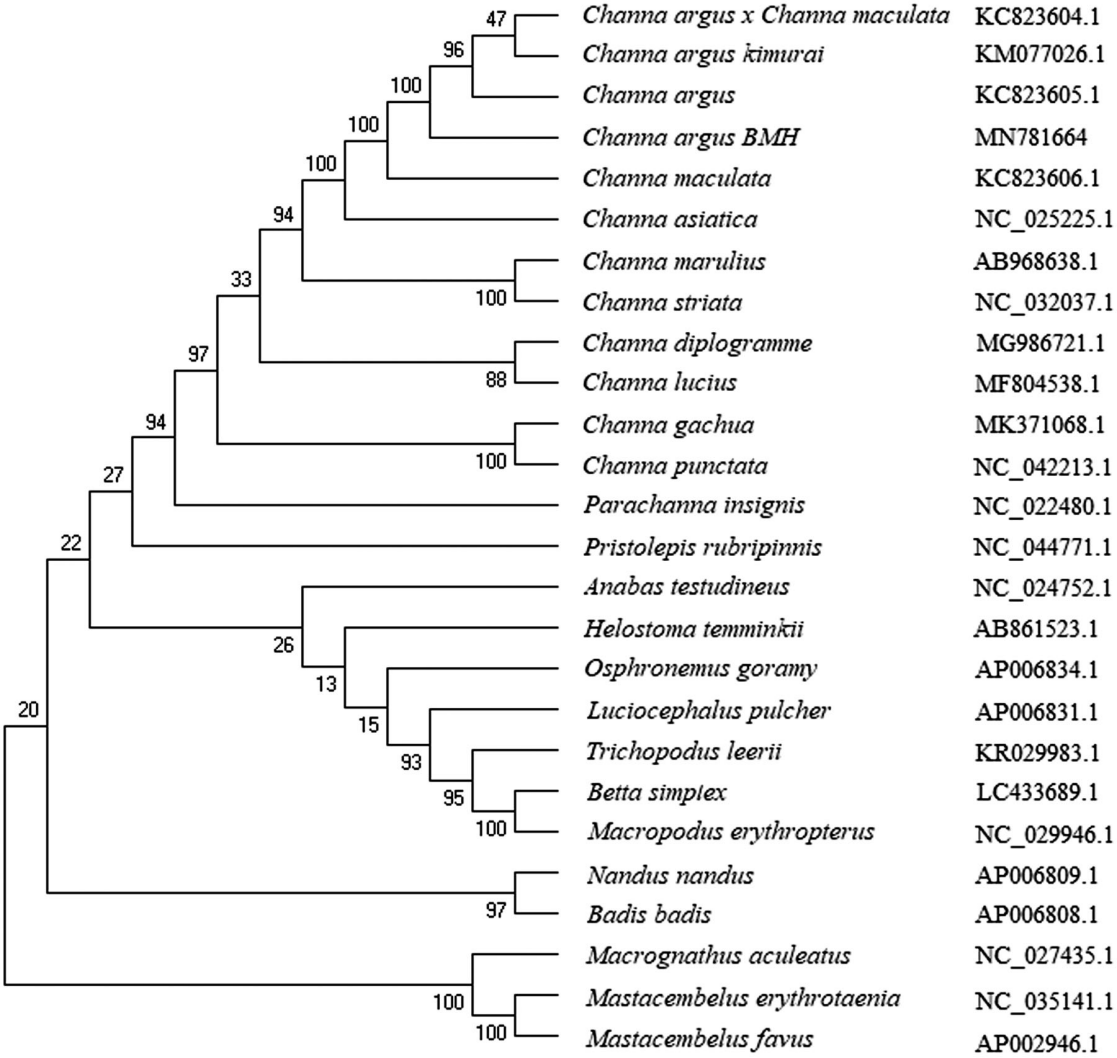
The phylogenetic analysis of *Channa argus* and other *Channa* fishes based on the mitogenome sequences.

## Data Availability

The data that support the findings of this study are openly available at NCBI (https://www.ncbi.nlm.nih.gov), GenBank accession no. MN781664. And the data that support the findings of this study are also available from the corresponding author, Dr. Yang, upon reasonable request.
